# DNA damage effects of inhalation anesthetics in human bronchoalveolar cells

**DOI:** 10.1097/MD.0000000000016518

**Published:** 2019-08-09

**Authors:** Zafer Cukurova, Halil Cetingok, Sukru Ozturk, Asuman Gedikbasi, Oya Hergunsel, Derya Ozturk, Burak Don, Kivanc Cefle, Sukru Palanduz, Devrimsel Harika Ertem

**Affiliations:** a Bakirköy Sadi Konuk Training and Research Hospital, Department of Anesthesiology and Intensive Care; b Istanbul University Medical Faculty, Department of Anesthesiology; c Istanbul University Medical Faculty, Department of Internal Medicine, Division of Medical Genetics, Bakirköy Sadi Konuk Training and Research Hospital, Biochemistry Lab; d University of Health Science, Sisli Hamidiye Etfal Research and Training Hospital, Department of Neurology, Istanbul, Turkey.

**Keywords:** 8-hydroxy-2-deoxyguanosine, bronchoalveolar cells, comet assay, desflurane, DNA damage, inhalation anesthetics, sevoflurane

## Abstract

**Background:**

The main objective was to evaluate and compare the local genotoxicity of sevoflurane and desflurane in bronchoalveolar cells, while the secondary outcome was to detect systemic oxidative DNA damage. To our knowledge, our study is the first one to evaluate the local effects of inhalation anesthetics in human bronchoalveolar cells in patients.

**Methods:**

American Society of Anesthesiologists group I-II patients scheduled for lumbar discectomy surgery were enrolled in this randomized prospective study. Patients were randomized to sevoflurane or desflurane for anesthesia maintenance. Bronchoalveolar lavage samples and peripheral blood samples were taken at 2-time points: the first point (baseline, T1); and the second point (postexposure, T2). Final number of 48 samples were the sevoflurane (n = 22) and desflurane (n = 26) groups. Comet assay was applied to examine genotoxic properties. Oxidative DNA damage in plasma was measured with 8-hydroxy-2′-deoxyguanosine (8-OHdG).

**Results:**

T2 values were higher than baseline values in both the desflurane group (tail-length: 66 ± 24, %DNA in tail: 72 ± 60, tail moment: 47.52 ± 14.4; *P* = .001, *P* = .005, *P* = .001, respectively) and the sevoflurane group (tail-length: 58 ± 33, %DNA in tail: 88 ± 80, tail moment: 51.04 ± 26.4; *P* = .001, *P* = .012, *P* = .001, respectively). T2 plasma 8-OHdG levels were also higher than baseline levels in the desflurane group (3.91 ± 0.19 ng/ml vs 1.32 ± 0.20 ng/ml, *P* = .001) and sevoflurane group (3.98 ± 0.18 ng/ml vs 1.31 ± 0.11 ng/ml, *P* = .001). There were no differences between the 2 groups in comet parameters and 8-OHdG levels.

**Conclusion:**

Our results indicate that both inhalation agents cause DNA damage in the bronchoalveolar cells. Also, we detected increases in plasma 8-OHdG concentrations. Local genotoxicity and systemic oxidized DNA damage were similar in both groups.

## 1 Introduction

Genotoxicity refers to a damaging effect on the genetic material including single-strand breaks, double-strand breaks, alkali labile sites, and DNA adducts (a covalent binding between a substance and DNA) induced by physical, biological, or chemical agents. Unless the damage to the genetic material can be repaired, DNA sequence alterations, single or multiple nucleotide changes that can lead to chromosomal aberration may occur. Recombination, mutation, tissue damage, aging, and cancer may develop as a result of these changes.^[1]^

The single-cell gel electrophoresis or comet assay is a sensitive, powerful, and safe method for detecting DNA strand breaks, which are a significant indicator of genotoxic and cytotoxic effects on cells caused by chemical and physical factors.^[2]^ The comet assay is commonly utilized to assess DNA damage in individual cells both in vitro and in vivo. It has proved its usefulness and versatility in human biomonitoring, ecogenotoxicology, genotoxicity testing, and basic research into the mechanisms of DNA damage and repair.^[3]^ The working group of the 6th International Workshop on Genotoxicity Testing (IWGT) focused solely on the in vivo comet assay and its use in regulatory genotoxicity testing.^[4]^ The in vivo comet assay is able to detect DNA damage in any tissue, even those with nonproliferating cells, and its high sensitivity makes it especially beneficial for the detection of genotoxicity. In vitro comet assay (cell culture) allows the screening of large numbers of genotoxic compounds.^[5]^ DNA damage is detected at the single-cell level using micro-gel technique including electrophoresis in alkaline (pH >13) conditions. The assay depends on relaxation of supercoiled loops by strand breaks; only those loops that are relaxed are able to move into a tail under electrophoresis, which often resembles a comet, observed by fluorescent microscope.^[6,7]^

Sevoflurane (fluoromethyl 2,2,2-trifluoro-1-(trifluoromethyl) ethyl ether) and desflurane (1,2,2,2-tetrafluoroethyl-difluoromethylether) are halogenated ethers and they are quite different from conventional volatile agents due to their low solubility. The blood/gas partition coefficient is a function of solubility of the agent in blood, and both of these agents have a lower blood/gas partition coefficient than other halogenated ethers.^[8]^ Clinical studies have verified that concentrations of sevoflurane and desflurane can be easily regulated and their recovery from anesthesia is rapid.^[9,10]^ Although sevoflurane was first synthesized in the 1970s, it was not used in clinical practice until the end of 1993 due to questions regarding its metabolism to 2% to 5% inorganic fluoride and the extent of sevoflurane degradation in the presence of CO_2_ absorbents.^[11]^ These carbon dioxide absorbents can degrade to compounds A, B, C, D, and E (an isomer of Compound D). Compound A can interact with nuclear DNA because of its highly reactive nature and alkylating activity.^[12]^ Desflurane is fluorinated methyl ether differing from isoflurane only in the substitution of fluorine for chlorine on the a-ethyl carbon. Fluorination increases the molecular stability and thus reduces the toxicity.^[13]^

There are studies in the literature examining the systemic genotoxic effects of similar anesthetic agents in peripheral blood cells.^[14–18]^ Although bronchoalveolar cells are the first to get in contact with the inhalation anesthetics, data showing the effects of inhalation anesthetics at cellular level is insufficient. We found only a few studies conducted by the same researchers evaluating local genotoxic effects of different anesthetic agents (halothane and penthrane) in bronchoalveolar and lung cells during in vitro exposure.^[19,20]^ They showed that both anesthetics provoked DNA fragmentation in bronchial epithelial cells and halothane exerts genotoxic and cytotoxic effect on the alveolar cells in vitro. In other study, researchers evaluated the genotoxicological effects of isoflurane in the lymphocytes and organs of rats. Their study has demonstrated that isoflurane exposure results in significant DNA damage in rat lymphocytes, bone marrow, spleen, brain, livers, and lung.^[21]^ Bronchoalveolar lavage (BAL) samples include cellular and acellular components of the distal bronchioles and gas exchange units. Bronchoalveolar cells play important roles in host defense, inflammation, and regulation of immune responses.^[22]^ Determining the local genotoxic effects of inhalation anesthetics on bronchoalveolar cells would be helpful to better understand the risk of DNA damage. To our knowledge, our study is the first one to evaluate the local effects of inhalation anesthetics in human bronchoalveolar cells in patients.

The safety of anesthesia can be determined by its impact on oxidative stress and inflammation and various biomarkers have been developed for this purpose. For example, oxidative stress-induced DNA double-strand breaks can be detected by upregulation of 8-hydroxy-2-deoxyguanosine (8-OHdG).^[23]^ 8-OHdG is generated after the repair of reactive oxygen species-mediated DNA damage and is, therefore, one of the most widely recognized biomarkers of oxidative damage of DNA.^[24]^ Secondarily, we evaluated plasma (8-OHdG) level is an indicator of the balance between oxidative DNA damage and repair mechanisms in the systemic circulation.^[25]^

Thus, the aim of this study was to investigate DNA damage in bronchoalveolar cells using comet assay in order to determine the local effects of the 2 most commonly used inhalation anesthetics. The main objective was to evaluate and compare sevoflurane and desflurane in terms of their genotoxicity in bronchoalveolar cells, and the secondary objective was to detect systemic oxidative DNA damage.

## 2 Methods

This study was a randomized self-controlled prospective clinical study. Following ethics committee approval (protocol no: 2012/92) and in accordance with the Declaration of Helsinki, the study included 54 American Society of Anesthesiology (ASA) Class I or II patients between 18 and 65 years of age who were scheduled for lumbar discectomy surgery. The duration of anesthesia was at least 60 minutes.

Patients with ASA Class 3 or 4 disease, malignancy, or chronic pulmonary disorders and those who required blood transfusion were excluded. Smokers, alcoholics, obese subjects, and those who had recently received radiation, medications, and/or antioxidant supplements were excluded from the study. Patients with known occupational exposures (operating room personnel, chemical plant workers) were also excluded. All patients signed a consent form before surgery. Patients were randomly allocated into the 2 groups according to the protocol number given from the hospital information system was odd number or even number: sevoflurane group (27) and desflurane group (27). The samples were coded with patient protocol number at 2-time points; first sample “T1” and second sample “T2.” Six patients (5 sevoflurane and 1 desflurane) were excluded from the study because their BAL samples that did not have sufficient living cells for comet analysis, resulting in a final number of 48 patients between the sevoflurane (n = 22) and desflurane (n = 26) groups.

### 2.1 General anesthesia

In preoperative preparation rooms, intravenous midazolam (1.5 mg) was given for sedation. Electrocardiogram, arterial blood pressure (systolic, diastolic, and mean), heart rate, and peripheral oxygen saturation were monitored and recorded at 10-minute intervals during surgery for all patients. General anesthesia was induced with 1.5 mcg/kg fentanyl, 2 mg/kg propofol, and 0.6 mg/kg rocuronium. After endotracheal intubation, patients received either 2% sevoflurane or 6% desflurane plus remifentanil infusion at 0.01 to 0.1 mcg/kg/min for maintenance of anesthesia. The lungs were mechanically ventilated using the volume-controlled mode with a tidal volume of 6 ml/kg and respiratory rate of 10 to 15 breaths/min to maintain an end-tidal carbon dioxide tension between 30 and 35 mm Hg. Fresh gas flow rate with FiO_2_ 50% was adjusted to 3 liters per minute. N_2_O was not used in order to avoid any possible additional DNA damage in the patients. Intraoperative normothermia (>35.0°C) was maintained using forced air warming devices (Covidien WarmTouch, model WT-5800, Covidien llc, 15 Hampshire Street, Mansfield, MA) with a specific blanket on the lower limbs, set to deliver forced-air at 42 to 46C following prone position. Postoperative pain relief was provided by a intravenous patient-controlled analgesia device delivering morphine 20 to 40 μg/kg at 10-minutes lockout intervals, after the last sampling. No routine medication was used for postoperative nausea and vomiting prophylaxis.

### 2.2 Sampling

**Figure 1:**
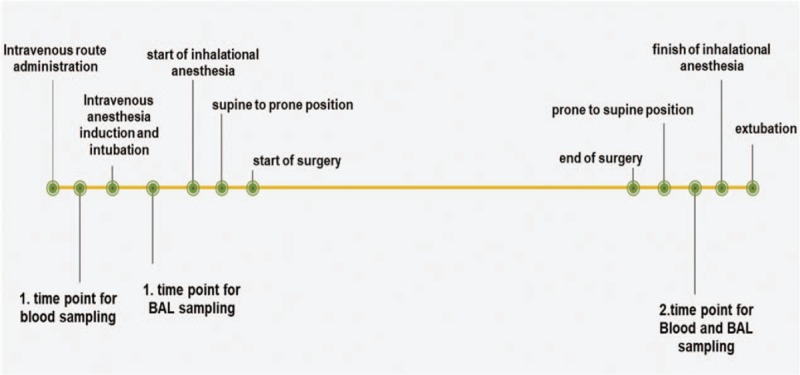
Sampling time point graphic.

The first peripheral blood was taken before the anesthesia (baseline; T1 blood). After intubation, the first BAL sample was taken (baseline; T1 BAL). The second BAL and peripheral blood samples were taken at the end of surgery (postexposure; T2 blood and T2 BAL) (Fig. 1). BAL samples were obtained using 30 ml of saline in sterile conditions (Muco-Safe w. Filter; Unomedical, Convatec Limited, CH5 2NU, UK) and were immediately taken to the laboratory. Peripheral blood samples were obtained from upper extremity veins following local disinfection with betadine. Peripheral blood samples (2 ml) were transported to the laboratory in tubes (BD-Plymouth. PL6 7BP.UK) containing lithium/heparin (68 IU).

### 2.3 Comet analysis

**Figure 2:**
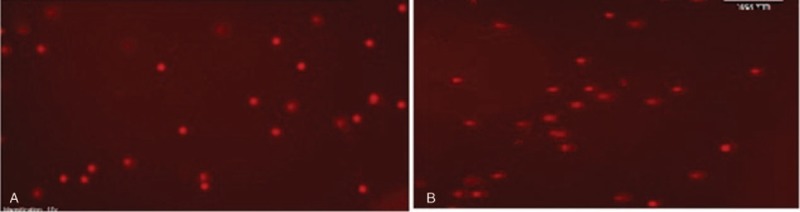
Evaluation of DNA damage by comet assay. (A) Microscopic image of baseline (T1) bronchoalveolar cells; (B) Microscopic image of post-exposure (T2) bronchoalveolar cells showing moderate DNA damage (200×).

BAL material was immediately centrifuged at 4°C for 10 minutes. The supernatant was removed and phosphate-buffered saline was added on the pellet to bring the volume to 1 ml. Viability test was performed in order to confirm the presence of adequate living cells. For this purpose, 10 μl of sample was mixed with the same amount of trypan blue stain and counted on Thoma slide. Samples showing approximately 15.000 living cells per 10 μl were included in our study (bright and nonstained cells are viable). The cell viabilites were >80% in 2 groups. At this stage, 6 samples that did not have sufficient cells for analysis were excluded from the study. The protocol used followed the general procedures described by Singh et al^[26]^ and Tice et al^[27]^ with some modifications. Firstly, 500 μl of 1% agarose was spread onto the slide and cooled. Then, 75 μl of 1% low-melting-point agarose and 20 μl of sample were mixed and spread onto the slides. Slides were incubated at 4°C for a couple of minutes, and then in lysis solution for 4 hours. After 20 minutes in alkaline electrophoresis solution, samples were run in electrophoresis (300 A, 25 V) for 15 minutes in a cool environment. All steps of the technical experiments were performed under dim yellow light to prevent further induction of DNA damage. After electrophoresis, slides were washed with phosphate-buffered saline and stained for 10 minutes with 20 μg/ml ethidium bromide. Comets were visualized with a Zeiss fluorescence microscope (Metasystem Isis) using to 200× magnification. Images from 100 nucleoids (50 from each replicate slide) per time point per patient were scored using TriTek Comet Slide v1.5 Freeware software (Fig. 2). DNA damage was expressed as Arbitrary Units (AU) based on pixel intensity. Of the 17 parameters provided by the program, we selected tail length, % DNA in the tail (tail intensity), and tail moment to estimate the extent of DNA damage. Cells with the core completely fragmented were not counted during analysis.

### 2.4 8-OHdG measurement

Blood samples collected at the 2-time points were centrifuged at 1000 g for 15 minutes. Plasma was removed and stored frozen at −70°C until analyzed. Plasma 8-OHdG assays were performed using competitive enzyme-linked immunosorbent assay (ELISA) from NWLSS 8-OHdG ELISA Kit Northwest (Vancouver, WA, Canada). The lower limit of detection was 0.125 ng/ml. The results are expressed in ng/ml.

### 2.5 Statistical methods

Statistical calculations were performed with NCSS (Number Cruncher Statistical System) 2007 software (Utah) program for Windows. Standard descriptive statistical calculations were expressed as mean ± standard deviation. For the variables indicate a normal distribution; unpaired *t* test was used in the comparison of groups and paired *t* test was employed in the assessment of baseline and postexposure values. For the variables does not indicate a normal distribution; Mann–Whitney *U* test was used in the comparison of groups and Wilcoxon test was employed in the assessment of baseline and postexposure values. Chi square test was performed during the evaluation of qualitative data. Statistical significance level was established at *P* < .05.

In the power analysis of our study, the difference in Tail intensity between groups was found as 11% to 38% (with a probability of alpha error = 0.05) carried out by using G-power 3.1 program; the sample size calculation has revealed that the required subject number was found 22, assuming value for power is 0.8.^[28]^ We increased by 20% to accommodate missing data.

## 3 Results

Twenty-six patients were included in the desflurane group and 22 in the sevoflurane group. There were no statistically significant differences in mean age, sex distribution, and the mean surgery duration between the sevoflurane and desflurane groups (*P* > .05) (Table 1). There was also no significant difference in the hemodynamic parameters (mean arterial pressure and heart rate) between 2 groups (*P* > .05) (Table 2). With regard to comet parameters, *T*_1_ (baseline) and *T*_2_ (postexposure) values were significantly different in both groups. Comet parameters (tail length, % DNA in tail, tail moment) showed significantly higher values at *T*_2_ compared to baseline levels (*P* < .05 for desflurane and *P* < .05 for sevoflurane). However, there were no statistically significant differences between the 2 groups in *T*_1_ and *T*_2_ values of comet parameters (*P* > .05) (Table 3) The ranges of tail length, % DNA in Tail and tail moment were given as AU in this study. Plasma 8-OHdG levels were also higher at *T*_2_ than at baseline in both the desflurane group and sevoflurane group (*P* < .05) and did not differ significantly between the 2 groups at either time point (*P* > .05) (Table 4).

**Table 1:**
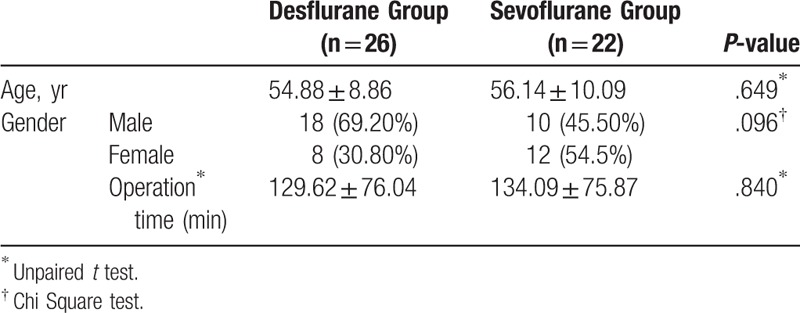
Comparison of the demographic and intraoperative characteristics. The data are shown in mean and standard deviation.

**Table 2:**
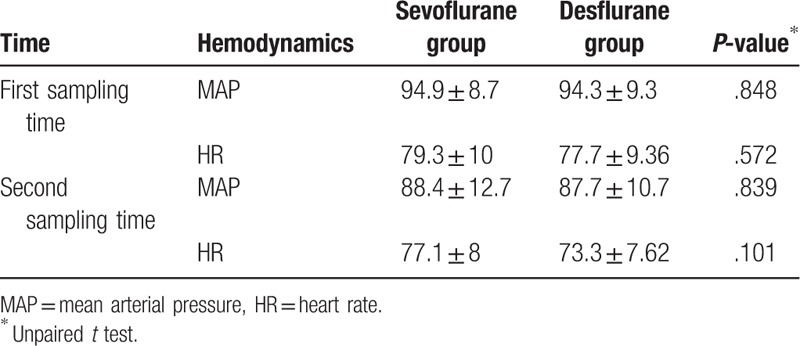
Comparison of the hemodynamic parameters (the data are shown in mean and standard deviation).

**Table 3:**
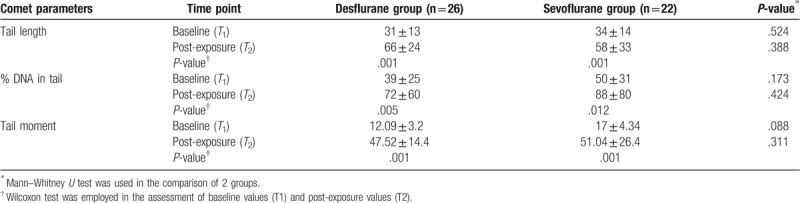
Comparison of the comet parameters of bronchoalveolar cells in baseline (T1) and post-exposure (T2) periods of both drug groups (the data are shown in mean and standard deviation). The ranges of tail length, %DNA in tail and tail moment were given as arbitrary unit in this table.

**Table 4:**
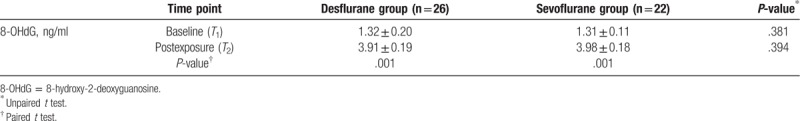
Comparison of the baseline and post-exposure plazma 8-OHdG levels in desflurane and sevoflurane group (the data are shown in mean and standard deviation).

## 4 Discussion

The systemic genotoxic effects of inhalation anesthetics have been reported in previous studies evaluating DNA damage in peripheral leukocytes as in vivo or in vitro (in cell culture).^[14–18,27–33]^ Also, there is a study evaluating local genotoxic effects of halothane and penthrane in bronchoalveolar cells during in vitro exposure.^[19–20]^ In this study, the local effects of the 2 most commonly used inhalation agents were investigated on bronchoalveolar cells in patients. We analyzed DNA damage using comet method to estimate local genotoxic effect of inhalation anesthesia with sevoflurane and desflurane on bronchoalveolar cells in patients.

During case selection for this study, we avoided severe diseases (diabetes, hypertension, cardiovascular diseases, cancer, etc) that may augment effects of DNA damage. Therefore, only patients scheduled for elective surgery for spine (lumbar discectomy surgery) were included in the study. Patients without systemic disease were the majority in this group. Anesthetic exposure time of the bronchoalveolar epithelium was the main parameter affecting the study outcomes. However, we could not fix the second sampling time because we could not get the expected operation time from the surgical team. The duration of these surgeries was at least 60 minutes. A fixed time could not be established for taking the second sample due to technical difficulties. To take the second sample, the BAL procedure was planned for the end of the operation, when the patients switched from prone position to supine position. However, when the durations of operations were compared, there was no statistically significant difference between groups in terms of duration of anesthesia.

Previous studies investigating whether inhalation anesthetics cause DNA damage in peripheral blood cells have yielded conflicting results. Orosz et al^[14]^ evaluated possible toxic effects of balanced anesthesia maintained with sevoflurane and showed that sevoflurane appears neither to damage DNA nor to alter redox status. Karabiyik et al^[17]^ evaluated the genotoxic properties of sevoflurane in human lymphocytes using in vivo comet assay. The authors reported detecting DNA damage due to sevoflurane, but concluded that cellular DNA repair occurred within 5 days. Alleva et al^[29]^ evaluated the genotoxicity of sevoflurane on DNA of lymphocytes and reported that the risk caused by sevoflurane was considerably low. Szyfter et al^[32]^ conducted a study of sevoflurane genotoxicity in vivo and in vitro conditions by using the comet assay through comparison with halothane and isoflurane. They concluded that sevoflurane was not genotoxic in vivo or in vitro. Otherside Brozovic et al^[34]^ evaluated the DNA damage and repair in kidney cells of mice after repeated exposure to sevoflurane and isoflurane and showed that sevoflurane was slightly more genotoxic than isoflurane. Lüleci et al^[31]^ showed that sevoflurane administration may affect cell division and have a mutagenic effect on DNA. Karpinski et al^[33]^ demonstrated that the genotoxicity of desflurane was capable of increasing DNA migration in a dose-dependent manner under experimental conditions applied. Nogueira et al^[15]^ showed with the comet assay that surgical patients anesthetized with desflurane (6%) had increased damage of lymphocyte DNA. Akin et al^[18]^ revealed that exposure to desflurane increased sister chromatid exchange in human lymphocytes, thereby this agent may evoke genetic damage.

Our study showed that sevoflurane and desflurane induce strand breaks or alkali-labile sites in bronchoalveolar cells from patients undergoing lumbar discectomy surgery. DNA single-strand breaks demonstrated by comet assay can indicate damage before DNA repair complete. Therefore, detected DNA single-strand breaks may be reversible. In addition the types of surgeries could have influenced the results. Also, the types of surgeries along the drugs used during surgeries and the halogenated anesthetics could influenced the findings. Those issues couldn’t be separated in this study. However, DNA repair is not always successful and an increased number of DNA single-strand breaks could lead to irreversible DNA damage.^[30]^ We did not observe differences regarding local genotoxicity and systemic oxidized DNA damage when comparing patients under sevoflurane or desflurane. In our study, the similar genotoxic effect of sevoflurane and desflurane can be explained by the similarity of chemical structures.

Previous studies have demonstrated that 8-OHdG formation is correlated with DNA modifications. This formation is independent of reactive oxygen species type (peroxides, superoxide, hydroxyl radical, etc) and exposure time.^[35]^ Jaloszynski et al^[36]^ hypothesized that polyfluorinated anesthetics can alkylate the N-7 position of purines. They believe that anesthetic genotoxicity might also be due to their metabolic oxidation or reduction, giving rise to reactive metabolites and reactive oxygen species. To evaluate systemic DNA damage induced by oxygen radicals in the present study, we measured 8-OHdG levels. We opted to use the comet method for cell-level analysis because our primary objective was to investigate damage to bronchoalveolar cell DNA locally induced by inhalation anesthetics. However, we preferred plasma 8-OHdG analysis to assess the systemic situation because it has been established as a valid marker of the equilibrium between DNA damage and repair mechanisms.^[23,37]^ In our study, we observed increases in plasma 8-OHdG concentrations as a marker of oxidative DNA damage with both desflurane and sevoflurane. Also, the study showed that sevoflurane and desflurane may induce DNA damage effects on bronchoalveolar cells.

One limitation of our study was that a fixed time could not be established for taking the second sample due to technical difficulties. The BAL procedure was planned for the end of the operation, when the patients switched from prone position to supine position for access to adequate lavage material. The other limitation of our study is that BAL is an invasive procedure and is performed twice during the course of our study (before and immediately after anesthesia), but the DNA damage associated with this procedure could not be assessed.

In conclusion, our study indicates that inhalation anesthetics may exert genotoxic effects on bronchoalveolar cells. Also, we observed increases in plasma 8-OHdG concentrations in both groups. There were no differences regarding local genotoxicity and systemic oxidized DNA damage when comparing patients under sevoflurane or desflurane.

## Acknowledgment

The authors wish to thank Ms. Rana Konyalioglu for her statistical expertise and helpful suggestions.

## Author contributions

**Conceptualization:** Zafer Cukurova, Halil Cetingok.

**Data curation:** Zafer Cukurova, Halil Cetingok, Oya Hergunsel.

**Formal analysis:** Zafer Cukurova, Halil Cetingok.

**Funding acquisition:** Zafer Cukurova, Halil Cetingok, Asuman Gedikbasi.

**Investigation:** Zafer Cukurova, Halil Cetingok, Asuman Gedikbasi, Oya Hergunsel, Kivanc Cefle.

**Methodology:** Zafer Cukurova, Halil Cetingok, Asuman Gedikbasi, Oya Hergunsel, Burak Don, Kivanc Cefle, Devrimsel Harika Ertem.

**Project administration:** Zafer Cukurova, Halil Cetingok, Sukru Ozturk, Asuman Gedikbasi, Derya Ozturk, Burak Don, Kivanc Cefle, Sukru Palanduz.

**Resources:** Zafer Cukurova, Asuman Gedikbasi, Derya Ozturk, Burak Don, Kivanc Cefle, Sukru Palanduz.

**Software:** Asuman Gedikbasi, Derya Ozturk, Burak Don, Kivanc Cefle, Sukru Palanduz.

**Supervision:** Sukru Ozturk, Asuman Gedikbasi, Kivanc Cefle, Sukru Palanduz, Devrimsel Harika Ertem.

**Validation:** Asuman Gedikbasi, Kivanc Cefle, Devrimsel Harika Ertem.

**Visualization:** Halil Cetingok, Sukru Ozturk, Devrimsel Harika Ertem.

**Writing – original draft:** Halil Cetingok, Sukru Ozturk, Asuman Gedikbasi, Devrimsel Harika Ertem.

**Writing – review and editing:** Halil Cetingok, Sukru Ozturk, Asuman Gedikbasi, Devrimsel Harika Ertem.

## References

[1] Nofer TW Nofer JR Jajte J Oxidation damage to DNA and oxidative stress in subjects occupationally exposed to nitrous oxide (N2O). Mutat Res 2012;731:58–63. 22085808 10.1016/j.mrfmmm.2011.10.010

[2] Collins AR The comet assay for DNA damage and repair: principles, applications, and limitations. Mol Biotechnol 2004;26:249–61. 15004294 10.1385/MB:26:3:249

[3] Collins AR The comet assay: a heavenly method. Mutagenesis 2014;30:1–4. 10.1093/mutage/geu079 25527721

[4] Speit G Kojima H Burlinson B Critical issues with the in vivo comet assay: a report of the comet assay working group in the 6th International Workshop on Genotoxicity Testing (IWGT). Mutat Res Genet Toxicol Environ Mutagen 2015;783:6–12. 25953395 10.1016/j.mrgentox.2014.09.006

[5] Bajpayee M Kumar A Dhawan A The comet assay: assessment of in vitro and in vivo DNA damage. Methods Mol Biol 2013;1044:325–45. 23896885 10.1007/978-1-62703-529-3_17

[6] Speit G Rothfuss A The comet assay: a sensitive genotoxicity test for the detection of DNA damage and repair; Bjergbæk L - DNA repair protocols. Methods Mol Biol 2012;920:79–90. 22941597 10.1007/978-1-61779-998-3_6

[7] Tice RR Agurell E Anderson D Single cell gel/comet assay: guidelines for in vitro and in vivo genetic toxicology testing. Environ Mol Mutagen 2000;35:206–21. 10737956 10.1002/(sici)1098-2280(2000)35:3<206::aid-em8>3.0.co;2-j

[8] Esper T Wehner M Meinecke CD Blood/gas partition coefficients for isoflurane, sevoflurane, and desflurane in a clinically relevant patient population. Anesth Analg 2015;120:45–50. 25393590 10.1213/ANE.0000000000000516

[9] White PF Tang J Wender RH Desflurane versus sevoflurane for maintenance of outpatient anesthesia: the effect on early versus late recovery and perioperative coughing. Anesth Analg 2009;109:387–93. 19608808 10.1213/ane.0b013e3181adc21a

[10] Magni G Rosa IL Melillo G A comparison between sevoflurane and desflurane anesthesia in patients undergoing craniotomy for supratentorial intracranial surgery. Anesth Analg 2009;109:567–71. 19608833 10.1213/ane.0b013e3181ac1265

[11] Smith I Nathanson M White PF Sevoflurane a long-awaited volatile anaesthetic. Br J Anaesth 1996;76:435–45. 8785147 10.1093/bja/76.3.435

[12] Kadioglu E Sardas S Erturk S Determination of DNA damage by alkaline halo and comet assay in patients under sevoflurane anesthesia. Toxicol Ind Health 2009;25:205–12. 19482915 10.1177/0748233709106445

[13] Jone RM Desflurane and sevoflurane: inhalation anaesthetics for this decade? Br J Anaesth 1990;65:527–36. 2248821 10.1093/bja/65.4.527

[14] Orosz JE Braz LG Ferreira AL Balanced anesthesia with sevoflurane does not alter redox status in patients undergoing surgical procedures. Mutat Res Genet Toxicol Environ Mutagen 2014;773:29–33. 25308703 10.1016/j.mrgentox.2014.07.007

[15] Nogueira FR Braz LG De Andrade LR Evaluation of genotoxicity of general anesthesia maintained with desfluranein patients under minor surgery. Environ Mol Mutagen 2016;57:312–6. 27062561 10.1002/em.22012

[16] Braz MG Braz LG Barbosa BS DNA damage in patients who underwent minimally invasive surgery under inhalation or intravenous anesthesia. Mutat Res 2011;726:251–4. 21944903 10.1016/j.mrgentox.2011.09.007

[17] Karabiyik L Şardaş S Polat U Comparison of genotoxicity of sevoflurane and isoflurane in human lymphocytes studied in vivo using the comet assay. Mutat Res 2001;492:99–107. 11377249 10.1016/s1383-5718(01)00159-0

[18] Akin A Ugur F Ozkul Y Desflurane anaesthesia increases sister chromatid exchanges in human lymphocytes. Acta Anaesthesiol Scand 2005;49:1559–61. 16223406 10.1111/j.1399-6576.2005.00779.x

[19] Tanya Topouzova, Hristova, Elena Stephanova. Inhalation Anesthetics Provoke Reorganization of Nuclear Components in Human Bronchial Cells. Proceedings of Balkan Scientific Conference of Biology (May 19–21, 2005, Plovdiv, Bulgaria) Section Developmental Biology 2005;522–530.

[20] Stephanova E Hristova TT Hazarosova R Halothane-induced alterations in cellular structure and proliferation of A549 cells. Tissue Cell 2008;40:397–404. 18508102 10.1016/j.tice.2008.04.001

[21] Kim H Oh E Im H Oxidative damages in the DNA, lipids, and proteins of rats exposed to isofluranes and alcohols. Toxicology 2006;220:169–78. 16442689 10.1016/j.tox.2005.12.010

[22] Meyer KC Raghu G Baughman RP An official American Thoracic Society clinical practice guideline: the clinical utility of bronchoalveolar lavage cellular analysis in interstitial lung disease. Am J Respir Crit Care Med 2012;185:1004–14. 22550210 10.1164/rccm.201202-0320ST

[23] Lee YM Song BC Yeum KJ Impact of volatile anesthetics on oxidative stress and inflammation. Biomed Res Int 2015;1–8. 10.1155/2015/242709 PMC4458520 26101769

[24] Halliwell B Whiteman M Measuring reactive species and oxidative damage in vivo and in cell culture: how should you do it and what do the results mean? Br J Pharmacol 2004;142:231–55. 15155533 10.1038/sj.bjp.0705776 PMC1574951

[25] Minno AD Turnu L Porro B 8-Hydroxy-2-deoxyguanosine levels and cardiovascular disease: a systematic review and meta-analysis of the literature. Antioxid Redox Signal 2016;24:548–55. 26650622 10.1089/ars.2015.6508 PMC4827317

[26] Singh NP McCoy MT Tice RR A simple technique for quantitation of low levels of DNA damage in individual cells. Exp Cell Res 1988;175:184–91. 3345800 10.1016/0014-4827(88)90265-0

[27] Tice RR Andrews PW Hirai O The single cell gel (SCG) assay: an electrophoretic technique for the detection of DNA damage in individual cells. Adv Exp Med Biol 1991;283:157–64. 2068983 10.1007/978-1-4684-5877-0_17

[28] Oncul S Karabiyik L Coskun E Comparisons of the effects of the sevoflurane and propofol on acute ischemia reperfusion and DNA damages in rabbits. Rev Bras Anestesiol 2017;67:35–41. 27838115 10.1016/j.bjan.2016.10.001

[29] Alleva R Tomasetti M Solenghi MD Lymphocyte DNA damage precedes DNA repair or cell death after orthopaedic surgery under general anaesthesia. Mutagenesis 2003;18:423–8. 12960410 10.1093/mutage/geg013

[30] Szyfter K Szulc R Mikstacki A Genotoxicity of inhalation anaesthetics: DNA lesions generated by sevoflurane in vitro and in vivo. J Appl Genet 2004;45:369–74. 15306730

[31] Lüleci N Sakarya M Topçu I Effects of sevofluran on cell division and levels of sister chromatid exchange. Anasthesiol Intensivmed Notfallmed Schmerzther 2005;40:213–6. 15832240 10.1055/s-2005-861138

[32] Reitz M DasGupta K Brandt L Detection of DNA damage in stimulated human lymphocytes after enflurane exposure in vitro. Environ Res 1992;59:476–84. 1464295 10.1016/s0013-9351(05)80050-7

[33] Karpiński TM Poczekaj MK Stachecki I Genotoxicity of the volatile anaesthetic desflurane in human lymphocytes in vitro, established by comet assay. J Appl Genet 2005;46:319–24. 16110191

[34] Brozović G Oršolić N Rozgaj R Sevoflurane and isoflurane genotoxicity in kidney cells of mice. Arh Hig Rada Toksikol 2017;68:228–35. 28976881 10.1515/aiht-2017-68-2941

[35] Kanabrocki EL Ryan MD Murray D Circadian variation in multiple sclerosis of oxidative stress marker of DNA damage. A potential cancer marker? Clin Ter 2006;157:117–22. 16817500

[36] Jałoszyński P Kujawski M Wa̧sowicz M Genotoxicity of inhalation anesthetics halothane and isoflurane in human lymphocytes studied in vitro using the comet assay. Mutat Res 1999;439:199–206. 10023059 10.1016/s1383-5718(98)00195-8

[37] Valavanidis A Vlachogianni T Fiotakis C 8-Hydroxy-2’-deoxyguanosine (8-OHdG): a critical biomarker of oxidative stress and carcinogenesis. J Environ Sci Health C Environ Carcinog Ecotoxicol Rev 2009;27:120–39. 19412858 10.1080/10590500902885684

